# Simultaneous measurement of apalutamide and N-desmethylapalutamide in human plasma using high-performance liquid chromatography with ultraviolet detection

**DOI:** 10.1186/s40780-025-00489-4

**Published:** 2025-09-29

**Authors:** Toshinori Hirai, Kota Tsuge, Yasuyoshi Ishiwata, Keita Izumi, Kazutaka Saito, Masashi Nagata

**Affiliations:** 1 Department of Pharmacy, Institute of Science Tokyo Hospital, 1-5-45 Yushima, Bunkyo- ku, Tokyo, 113-8519 Japan; 2 Department of Urology, Dokkyo Medical University Saitama Medical Center, 2-1-50 Minamikoshigaya, Koshigaya city, 343-8555 Saitama Japan

**Keywords:** Apalutamide, N-desmethylapalutamide, High-performance liquid chromatography, Therapeutic drug monitoring

## Abstract

**Background:**

The combination of apalutamide, a nonsteroidal androgen receptor inhibitor, with androgen deprivation therapy enhances survival in patients with metastatic castration-sensitive prostate cancer. However, apalutamide exhibits complex pharmacokinetics and dose-dependent adverse effects, necessitating dose adjustments to optimize its therapeutic outcomes. To facilitate effective monitoring, a high-performance liquid chromatography (HPLC) system coupled with an ultraviolet (UV) detector was developed for quantifying plasma concentrations of apalutamide and its active metabolite, N-desmethylapalutamide.

**Main body:**

This method employed an ODS18 column (100 mm × 2.1 mm) with UV detection at 254 nm. The mobile phase comprised 20 mM acetate buffer (pH 5.0) and acetonitrile in a 60:40 ratio, and the run time was 10 min. The precision and accuracy were validated according to guidelines issued by the Food and Drug Administration (FDA). Long-term stabilities of the analytes were confirmed at both − 20 and − 80 °C over periods of 2 and 4 weeks. Peaks for enzalutamide (internal standard), N-desmethylapalutamide, and apalutamide were detected at 4.4, 5.8, and 7.7 min, respectively. Calibration curves demonstrated linearity within a concentration range of 0.5–20 µg/mL for both analytes in human plasma (*R*^2^ = 0.9999). Additionally, the intraday and interday variability and stability remained within FDA guidelines.

**Short conclusion:**

This work therefore presents a robust and simple HPLC–UV method for the simultaneous quantification of apalutamide and N-desmethylapalutamide in clinical therapeutic drug monitoring.

## Background

Apalutamide (4-[7-[6-cyano-5-(trifluoromethyl)pyridin-3-yl]-8-oxo-6-sulfanylidene-5,7-diazaspiro[3.4]octan-5-yl]-2-fluoro-*N*-methylbenzamide) is a nonsteroidal androgen receptor inhibitor (molecular weight, 477.43; pK_a,_ 9.7) [1]. Randomized controlled trials have demonstrated that combining apalutamide with androgen deprivation therapy enhances the overall survival in patients with metastatic castration-sensitive prostate cancer [2, 3]. Nonetheless, apalutamide frequently induces adverse effects such as skin rashes and fatigue [4, 5]. This toxicity compromises both quality of life and treatment intensity.

Prior research has indicated that the plasma concentration of apalutamide may influence its efficacy and toxicity profiles [6, 7], highlighting the necessity for dose adjustments. Apalutamide is metabolized primarily by cytochrome P450 (CYP) 2C8, and to a lesser extent by CYP3A4, into its active metabolite, N-desmethylapalutamide, which possesses approximately one-third of the antitumor activity exhibited by the parent drug [8]. To address interindividual variability, dosage should be personalized by considering both concentrations of apalutamide and N-desmethylapalutamide. However, a reliable method for measuring the concentrations of these compounds in human plasma has yet to be established. Therefore, the development of an analytical approach to quantify the plasma levels of apalutamide and N-desmethylapalutamide in clinical practice remains a significant challenge.

Androgen receptor inhibitors share similar chemical characteristics (e.g., pK_a_) and appropriate analytical methods have been established for several drugs (with the exception of apalutamide) using high-performance liquid chromatography (HPLC) combined with ultraviolet (UV) detection or tandem mass spectrometry [9]. As HPLC tandem mass spectrometry is not widely available in medical facilities, HPLC-UV serves as a practical alternative method.

This study therefore aims to develop a novel and practical HPLC-UV method for the simultaneous measurement of apalutamide and N-desmethylapalutamide levels in human plasma.

## Methods

### Chemicals

Apalutamide was sourced from Selleck Biotechnology Ltd. (Kanagawa, Japan), while N-desmethylapalutamide was obtained from MedChemExpress Ltd. (Illinois, USA). Enzalutamide, which was used as the internal standard (IS), was acquired from Funakoshi Co., Ltd. (Tokyo, Japan).

### Sample preparation

Stock solutions of apalutamide and N-desmethylapalutamide (300 µg/mL each) were prepared in methanol. These stock solutions were diluted to concentrations of 5, 10, 25, 50, 100, and 150 µg/mL using methanol to give the corresponding standard solutions. Enzalutamide was dissolved in acetonitrile to give a concentration of 4 µg/mL.

Calibration standards were also established at concentrations of 0.5, 1, 2.5, 5, 10, and 20 µg/mL for apalutamide and N-desmethylapalutamide by adding an arbitrary amount of drug-free plasma. Following preparation, an aliquot (100 µL) of each calibration standard and the IS solution (200 µL) was mixed in a microtube, vortexed for 10 s, and then centrifuged at 15,000 × *g* and 4 °C for 10 min. The resulting supernatant (150 µL) was diluted by adding 20 mM acetate buffer (pH 5.0, 100 µL) in a fresh microtube. After centrifugation at 10,000 g and 4 °C for 2 min, an aliquot (50 µL) of the supernatant was injected into the HPLC system.

### HPLC conditions

The HPLC system (Shimadzu Corporation, Japan) was equipped with a Kinetex 2.6 μm C18 column (100 mm length × 2.1 mm inner diameter, Phenomenex Inc., Torrance, CA, USA) and a UV-visible detector set to 254 nm (Shimadzu Corporation, Japan). The mobile phase consisted of 20 mM acetate buffer (pH 5.0) and acetonitrile in a 60:40 ratio. The flow rate was maintained at 0.3 mL/min and the column temperature was 40 °C. A detection time of 10 min was employed.

### Validation

The analytical method was validated for linearity, recovery rate, intraday variability, interday variability, and long-term stability in accordance with Food and Drug Association (FDA) guidelines for validation of analytical procedures for drug concentration [10]. The equations for relative standard deviation (RSD %; i.e., the precision) and relative error (RE %; i.e., the accuracy) were as follows:

$$\:RSD=\frac{standard\:deviation}{mean\:value}\times\:100$$

$$\:RE=\frac{measured\:value-theoretical\:value}{theoretical\:value}\times\:100$$

The mean RSD and RE values at each concentration were within ± 15% of the theoretical concentration across all levels, except at the lower limit of quantitation, where they were within ± 20%. To assess the stabilities of apalutamide and N-desmethylapalutamide, calibration standards in drug-free plasma were stored at − 20 and − 80 °C for 2 and 4 weeks. The ratio of the spiked values was calculated using the following equation:

$$\begin{gathered}\:Ratio\:of\:spiked\:values = \hfill \\\,\,\,\,\,\,\frac{{Spiked\:value}}{{Spiked\:value\:at\:0\:week}} \times \:100 \hfill \\ \end{gathered} $$

A ratio within ± 15% of the theoretical value at each concentration level was used as a criterion.

### Clinical application

The Institutional Review Board of Dokkyo Medical University Saitama Medical Center approved the study protocol (approval number: 24063). Written informed consent was obtained from patients treated with apalutamide. Prior to the subsequent dose, blood samples were collected at times of 0, 2, and 4 weeks after apalutamide administration. The patient received levodopa/carbidopa hydrate, safinamide mesylate, and atorvastatin.

## Results

### Selectivity and construction of the calibration curves

**Fig. 1:**
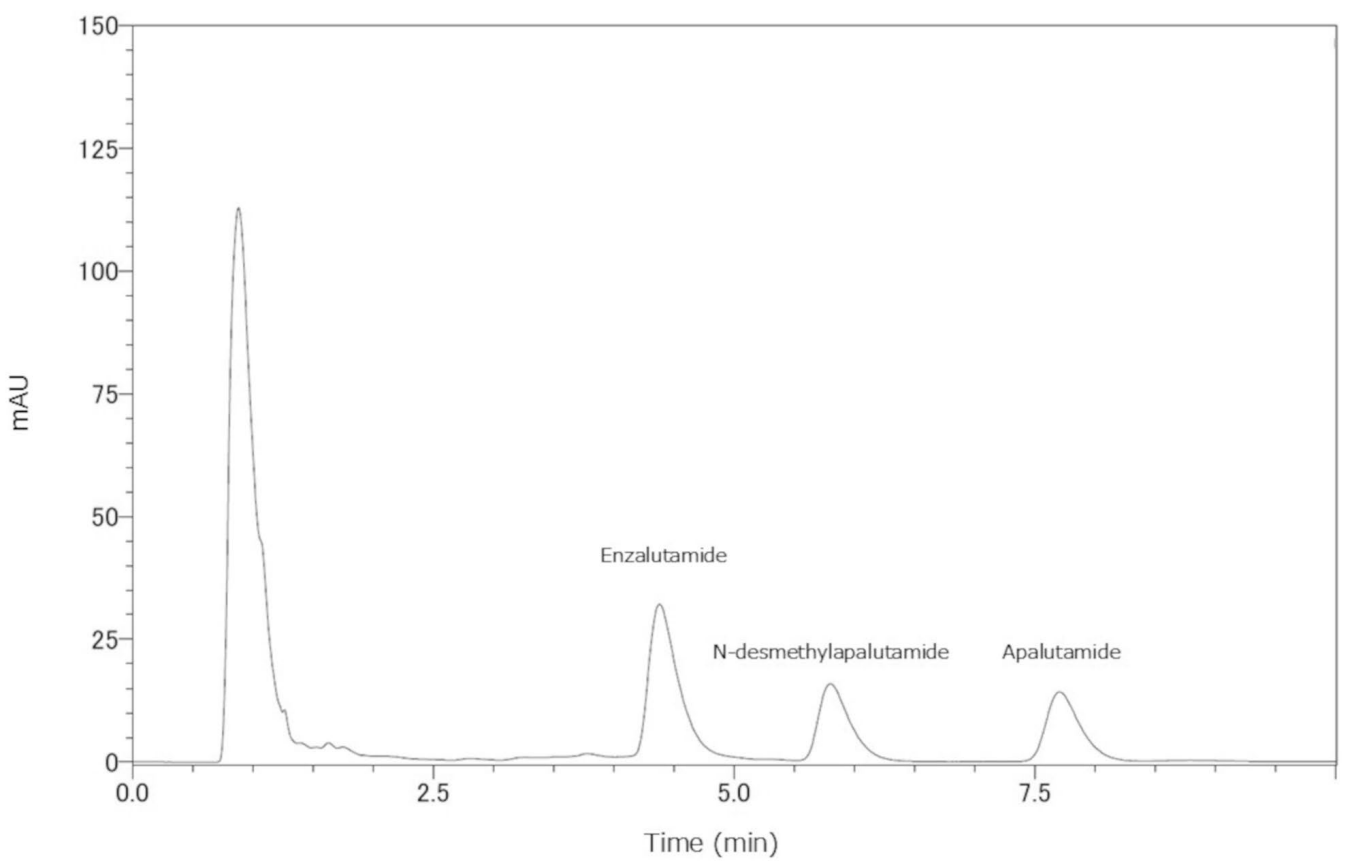
Chromatograms of apalutamide, N-desmethylapalutamide, and enzalutamide. The x-axis represents the retention time, while the y-axis represents the peak intensity. Enzalutamide was used as the IS. The concentrations are as follows: enzalutamide (4 µg/mL) and apalutamide and N-desmethylapalutamide (both 5 µg/mL)

**Fig. 2:**
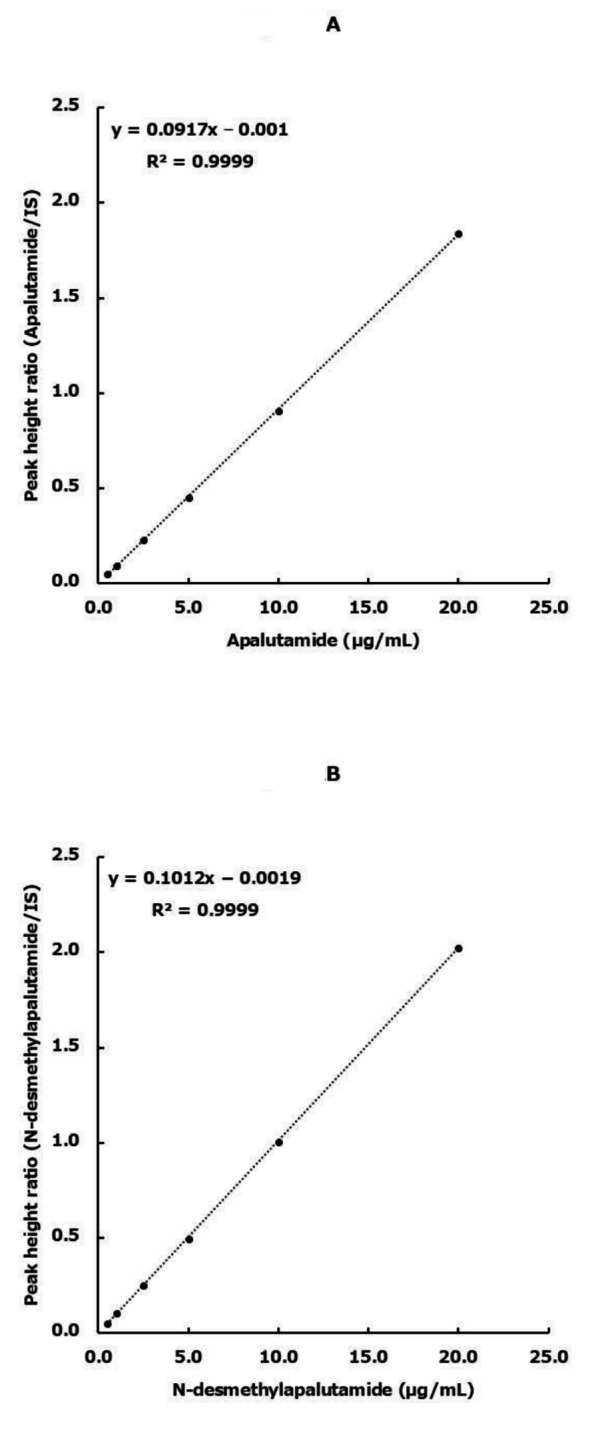
Calibration curves for apalutamide and N-desmethylapalutamide. **A**: Apalutamide: The x-axis represents the apalutamide concentration in the plasma, whereas the y-axis represents the peak height ratio of apalutamide to enzalutamide (IS). The dotted line indicates the calibration curve. **B**: N-desmethylapalutamide. The x-axis represents the N-desmethylapalutamide concentration in the plasma, whereas the y-axis represents the peak height ratio of N-desmethylapalutamide to enzalutamide (IS). The dotted line indicates the calibration curve

Figure 1 presents the chromatogram recorded for apalutamide and N-desmethylapalutamide at concentrations of 5 µg/mL. Peaks of enzalutamide, N-desmethylapalutamide, and apalutamide were observed with retention times of 4.4, 5.8, and 7.7 min, respectively. The calibration curve was linear between 0.5 and 20 µg/mL for both apalutamide and N-desmethylapalutamide in human plasma, and the lower limit of quantification for both compounds was 0.5 µg/mL. The coefficient of determination was 0.9999 for both analytes (Fig. 2A and B).

### Accuracy and precision

Intraday and interday variation of apalutamide and N-desmethylapalutamide met FDA criteria for both RSD and the RE (Table 1). The recovery rate ranges from 90 to 110%.

**Table 1:** Summary of the intraday and interday variabilities

Calibration solution	Recovery rate	Intraday variability, *n* = 5			Interday variability, *n* = 5		
µg/mL	%	Concentration, µg/mL	RSD, %	RE, %	Concentration, µg/mL	RSD, %	RE, %
Apalutamide							
0.5	92.1	0.53 ± 0.03	5.2	5.2	0.40 ± 0.06	16.0	−19.7
1	93.3	1.05 ± 0.03	2.7	5.2	0.97 ± 0.06	6.4	−2.5
2.5	100.7	2.56 ± 0.04	1.5	2.3	2.53 ± 0.07	2.7	1.1
5	98.8	4.94 ± 0.03	0.5	−1.2	5.11 ± 0.11	2.1	2.2
10	104.1	9.84 ± 0.11	1.1	−1.6	10.03 ± 0.31	3.1	0.3
20	102.8	20.09 ± 0.05	0.3	0.4	19.96 ± 0.15	0.7	−0.2
N-desmethylapalutamide							
0.5	96.9	0.54 ± 0.03	5.1	7.3	0.41 ± 0.06	13.9	−17.9
1	94.9	1.05 ± 0.03	3.2	5.1	0.97 ± 0.06	6.0	−2.7
2.5	100.7	2.56 ± 0.04	1.5	2.2	2.51 ± 0.07	2.8	0.6
5	98.9	4.94 ± 0.02	0.3	−1.2	5.11 ± 0.11	2.2	2.2
10	103.8	9.82 ± 0.12	1.2	−1.8	10.04 ± 0.3	3.0	0.4
20	103.1	20.09 ± 0.06	0.3	0.5	19.96 ± 0.14	0.7	−0.2

Data represent the mean ± standard deviation. RSD: relative standard deviation, RE: relative error

### Stability

The stability of apalutamide and N-desmethylapalutamide at each concentration adhered to FDA guidelines (Tables 2 and 3).

**Table 2:** Summary of the long-term storage test for apalutamide

	Spiked value, *n* = 3			Ratio of spiked value, %	
Calibration solution, µg/mL	0 week	2 weeks	4 weeks	2 weeks	4 weeks
−**20** °**C**					
0.5	1685 ± 55	1601 ± 84	1666 ± 23	95.0 ± 5.0	98.9 ± 1.3
1	3463 ± 84	3341 ± 15	3345 ± 89	96.5 ± 0.4	96.6 ± 2.6
2.5	8542 ± 74	8230 ± 263	8272 ± 54	96.3 ± 3.1	96.8 ± 0.6
5	17,456 ± 241	16,326 ± 332	16,824 ± 248	93.5 ± 1.9	96.4 ± 1.4
10	33,394 ± 1765	33,802 ± 657	33,257 ± 245	101.2 ± 2.0	99.6 ± 0.7
20	63,935 ± 1027	62,326 ± 1257	61,734 ± 2635	97.5 ± 2.0	96.6 ± 4.1
**−80 °C**					
0.5	1685 ± 55	1708 ± 19	1782 ± 44	101.3 ± 1.1	105.7 ± 2.6
1	3463 ± 84	3616 ± 115	3425 ± 78	104.4 ± 3.3	98.9 ± 2.3
2.5	8542 ± 74	8507 ± 366	8941 ± 186	99.6 ± 4.3	104.7 ± 2.2
5	17,456 ± 241	17,712 ± 360	17,002 ± 370	101.5 ± 2.1	97.4 ± 2.1
10	33,394 ± 1765	35,105 ± 806	34,488 ± 543	105.1 ± 2.4	103.3 ± 1.6
20	63,935 ± 1027	68,702 ± 752	67,750 ± 1408	107.5 ± 1.2	106.0 ± 2.2

Data represent the mean ± standard deviation

**Table 3:** Summary of the long-term storage test for N-desmethylapalutamide

	Spiked value, *n* = 3			Ratio of spiked value, %	
Calibration solution, µg/mL	0 week	2 weeks	4 weeks	2 weeks	4 weeks
−**20** °**C**					
0.5	1966 ± 16	2050 ± 76	2189 ± 12	104.3 ± 3.9	111.3 ± 0.6
1	3792 ± 65	3794 ± 18	3869 ± 86	100.1 ± 0.5	102.0 ± 2.3
2.5	9013 ± 154	8756 ± 290	8852 ± 65	97.2 ± 3.2	98.2 ± 0.7
5	18,292 ± 213	17,023 ± 357	17,608 ± 283	93.1 ± 2.0	96.3 ± 1.5
10	34,664 ± 2041	34,835 ± 676	34,335 ± 251	100.5 ± 1.9	99.1 ± 0.7
20	66,319 ± 1038	63,872 ± 1273	62,861 ± 2927	96.3 ± 1.9	94.8 ± 4.4
**−80 °C**					
0.5	1966 ± 16	2104 ± 23	2216 ± 29	107.0 ± 1.2	112.7 ± 1.5
1	3792 ± 65	4054 ± 149	3914 ± 97	106.9 ± 3.9	103.2 ± 2.6
2.5	9013 ± 154	9057 ± 432	9640 ± 210	100.5 ± 4.8	107.0 ± 2.3
5	18,292 ± 213	18,552 ± 383	17,873 ± 427	101.4 ± 2.1	97.7 ± 2.3
10	34,664 ± 2041	36,482 ± 986	35,959 ± 635	105.2 ± 2.8	103.7 ± 1.8
20	66,319 ± 1038	71,230 ± 944	70,586 ± 1763	107.4 ± 1.4	106.4 ± 2.7

Data represent the mean ± standard deviation

### Clinical application

**Fig. 3:**
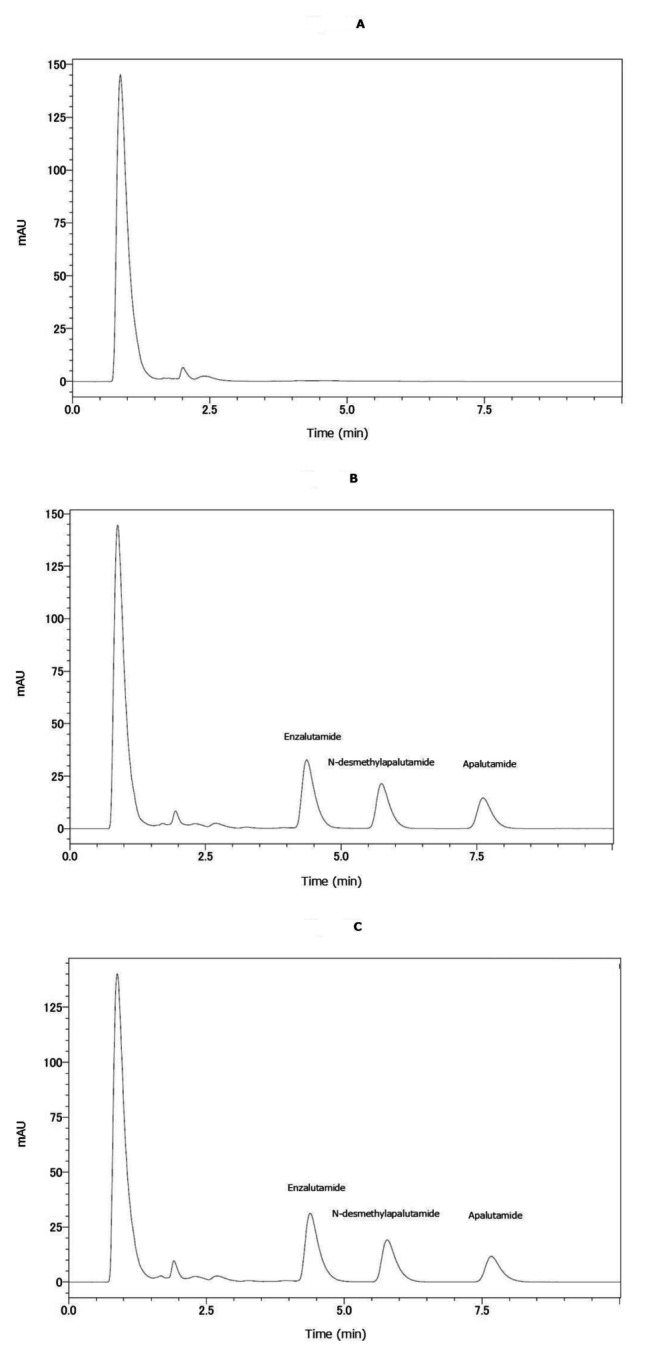
Chromatograms of apalutamide, N-desmethylapalutamide, and enzalutamide in plasma obtained from the patient. **A**: Prior to apalutamide administration. **B**: Two weeks after apalutamide administration. Enzalutamide was used as the IS. **C**: Four weeks after apalutamide administration. Enzalutamide was used as the IS. For each panel, the x-axis represents the retention time, whereas the y-axis represents the peak intensity

The chromatograms obtained for the samples collected from a representative patient receiving 240 mg of apalutamide once daily at 0, 2, and 4 weeks are depicted in Fig. 3A and B, and 3C, respectively. Spiked peaks were distinctly observed for enzalutamide, N-desmethylapalutamide, and apalutamide. These peaks were unaffected by either foreign substances or concomitant medications. After 2 weeks, the plasma trough concentrations of apalutamide and N-desmethylapalutamide were 5.45 and 7.13 µg/mL, respectively, with corresponding values of 4.57 and 6.66 µg/mL after 4 weeks. Similarly, no interference was observed from co-administered medications (Table 4).

**Table 4:** Summary of medications co-administered to patients

Patient	Medication
1	Tamsulosin, amlodipine, and lansoprazole
2	Levodopa/carbidopa, safinamide, and atorvastatin
3	Risedronate, nifedipine, telmisartan, and mosapride
4	Tramadol, acetaminophen, allopurinol, nifedipine
5	Mirogabalin, neurotropin, methylcobalamin, dapagliflozin, atorvastatin, telmisartan
6	Silodosin and fexofenadine

## Discussion

To the best of our knowledge, this work represents the first report of a straightforward simultaneous method for quantifying human plasma levels of apalutamide and N-desmethylapalutamide, using the HPLC-UV technique. The clear chromatogram was due to the relatively lipophilic mobile phase, and we believe that the first peak was formed by water-soluble substances in plasma. This method is expected to be a valuable tool for optimizing apalutamide dosing regimens through therapeutic drug monitoring.

The developed HPLC technique allows the measurement of apalutamide and N-desmethylapalutamide over a broader range of concentrations than previous methods, one of which demonstrated linearity for apalutamide in mice within a concentration range of 0.21–4.17 µg/mL [11]. In another study, trough steady-state concentrations of apalutamide and N-desmethylapalutamide were reported as 3.72 ± 1.19 and 4.66 ± 0.90 µg/mL, respectively [12]. Although liquid chromatography–tandem mass spectrometry offers a quantification range of 0.025–25.0 µg/mL [12], its availability is limited in many medical institutions. The developed HPLC-UV system, therefore, serves as a practical alternative for measuring apalutamide and N-desmethylapalutamide in human plasma. In this analysis, the calibration standards of apalutamide and N-desmethylapalutamide (i.e., 0.5–20 µg/mL) remained within the clinically observed concentration range of these compounds in plasma.

In the present study, use of HPLC was successfully validated, demonstrating acceptable intraday and interday variabilities. Long-term storage stability tests confirmed that the ratio of the spiked value for apalutamide remained within the range of 93.5 ± 1.9 to 107.5 ± 1.2%, while that for N-desmethylapalutamide remained within 93.1 ± 2.0 to 112.7 ± 1.5%. Additionally, this method offers the advantage of obtaining concentration data with shorter run times compared to the established retention times for apalutamide (13.6 min) and enzalutamide (11.4 min) [11]. The proposed measurement system will facilitate monitoring by clinicians due to its ability to effectively quantify the apalutamide and N-desmethylapalutamide levels in patient blood samples.

Based on previous research [13, 14], which indicates that apalutamide and N-desmethylapalutamide reach steady state levels ∼ 1–4 weeks post-administration owing to their extended half-life, our findings (Fig. 3A and B) largely corroborate this observation. Previously, significant inter-subject variability was observed relative to the reported mean trough concentration [12], underscoring the necessity for dose adjustments. Although patients received multiple concomitant medications that are commonly prescribed to patients with prostate cancer, these co-administered drugs did not interfere with the selectivity of the assay for the target compounds. Therefore, the proposed measurement system represents a valuable approach for therapeutic drug monitoring of apalutamide in clinical practice.

This study has some limitations. The stability of drug concentrations in patient plasma may exhibit differences owing to protein binding, metabolite interactions, and matrix-related factors that are not observed in spiked samples. Although a high injection volume was set to measure low concentration ranges when patient had reduced apalutamide due to adverse events, a broad peak due to large injection volume might interfere with quantification when co-administered with other drugs.

## Conclusions

In summary, the simplified HPLC-UV system developed herein can accurately quantify apalutamide and N-desmethylapalutamide in human plasma. Future studies will focus on the therapeutic drug monitoring of apalutamide in patients with prostate cancer using HPLC.

## Data Availability

The data that support the findings of this study are available from the corresponding author, N. M., upon reasonable request.

## Abbreviations

HPLC: High-performance liquid chromatography
UV: Ultraviolet
FDA: Food and Drug Administration
IS: Internal standard
